# Use of Computed Tomography in the Clinical Diagnosis of Lower Respiratory Tract Diseases in Sheep

**DOI:** 10.3390/vetsci12111070

**Published:** 2025-11-07

**Authors:** Enrique Castells, Pablo Quílez, Delia Lacasta, Aurora Ortín, Sergio Villanueva-Saz, María Climent, Lluís Luján, David Guallar, Carlos Alfonso Hedman, Héctor Ruiz, Marta Ruiz de Arcaute

**Affiliations:** 1 Centro Clínico Veterinario de Zaragoza, Madre Genoveva Torres Morales 8, 50006 Zaragoza, Spain; kikevet38@gmail.com; 2 Animal Pathology Department, Veterinary Faculty and Instituto Agroalimentario de Aragón-IA2, (Universidad de Zaragoza-CITA) University of Zaragoza, Miguel Servet 177, 50013 Zaragoza, Spain; pquilez@unizar.es (P.Q.); aortin@unizar.es (A.O.); svs@unizar.es (S.V.-S.); llujan@unizar.es (L.L.); 820880@unizar.es (D.G.); hedman@unizar.es (C.A.H.); hectorruiz353@gmail.com (H.R.); martarda@unizar.es (M.R.d.A.); 3 Ruminant Clinical Service, Veterinary Faculty, University of Zaragoza, Miguel Servet 177, 50013 Zaragoza, Spain; mariacli@unizar.es; 4 Department of Anatomy, Embryology, and Animal Genetics, Veterinary Faculty, University of Zaragoza, Miguel Servet 177, 50013 Zaragoza, Spain

**Keywords:** small ruminants, pulmonary lesions, diagnostic imaging, CT scan, lung pathology

## Abstract

**Simple Summary:**

Sheep play a vital role in food production and rural economies, but their health and welfare are often compromised by respiratory diseases. These conditions are difficult to differentiate on farms because many produce similar outward signs, such as coughing, weight loss, or breathing problems. As a result, treatment may be delayed or inadequate, and losses can increase. Computed tomography (CT) is an imaging tool widely used in human and companion animal medicine, but it has rarely been applied in sheep. This study explored whether CT could provide clearer information than traditional clinical methods when investigating lung problems in adult animals. The technique produced highly detailed images of the lungs, allowing different diseases to be distinguished more precisely and showing that some sheep were affected by more than one disorder at the same time. CT also gave additional clues about the stage and severity of the disease by measuring changes in lung tissue density. Although the method requires anaesthesia and may not yet be suitable for routine farm use, it offers valuable opportunities for research and specialist clinical cases. In the long term, improving diagnostic accuracy has the potential to benefit animal welfare, productivity, and the sustainability of sheep farming.

**Abstract:**

Lower airway diseases are a major health concern in sheep, often presenting with overlapping clinical signs that hinder accurate diagnosis. This study evaluated the diagnostic value of computed tomography (CT) in 58 adult sheep examined in northeastern Spain between 2017 and 2024. All animals underwent full clinical examination, CT under general anaesthesia, and post-mortem investigation. CT identified 82 pulmonary lesions, including interstitial pneumonia, respiratory complex, gangrenous pneumonia, caseous lymphadenitis, parasitic pneumonia, pulmonary adenocarcinoma, and pulmonary hydatidosis. Tissue density measurements provided additional information regarding disease stage and severity. The level of agreement between CT findings and definitive diagnoses consistently exceeded that of clinical evaluation, reaching almost perfect concordance for several conditions. Moreover, CT revealed concomitant respiratory pathologies within the same animal, many of which were not detected by conventional diagnostic methods. Although thorough individual clinical examination remains essential for the correct interpretation of imaging findings and the accurate diagnosis of respiratory disorders in the field, these results demonstrate that CT provides superior diagnostic accuracy compared with standard approaches and yields valuable insights for both clinical practice and research. Despite its practical limitations, CT could represent a major advance in improving health, welfare, and productivity in sheep farming.

## 1. Introduction

The global sheep industry is a vital part of the agricultural economy, supplying meat, milk, wool, and hides that generate significant local and international revenue. Its adaptability to diverse environments and low maintenance costs make it especially important in rural regions and developing countries, supporting food security and employment. Since 2000, the global sheep and goat population has grown steadily, driven by rising demand for meat and milk, with the most pronounced growth in Africa and Asia, Asia alone holding the largest share of the world’s population [1,2,3]. This growing demand has led to a progressive intensification of production systems, contributing to a higher incidence of health problems, particularly respiratory disorders. Both infectious and non-infectious diseases continue to pose a major challenge, causing substantial economic losses in the sheep industry, especially those affecting the respiratory system [4,5,6].

When investigating respiratory diseases in adult sheep, it is essential to distinguish between upper respiratory tract diseases (URDs) and lower respiratory tract diseases (LRDs), as these categories differ markedly in pathophysiology, clinical presentation, and management strategies [7]. This article focuses on LRDs, which in this study are considered to include conditions affecting the respiratory tract from the trachea to the pulmonary alveoli, in order to provide a more clinically oriented perspective. LRDs represent a significant health concern because of their potential to cause severe respiratory dysfunction, thereby compromising flock welfare and productivity [8]. The differential diagnosis of LRDs in adult sheep is often broad and complex, with classification commonly based on whether diseases are productive or non-productive according to clinical and pathological characteristics. However, many of these conditions share similar clinical signs, including chronic weight loss, dyspnoea, reduced productivity, and general debilitation, which complicates accurate diagnosis. Such clinical overlap can obscure the underlying aetiology and increase the risk of misclassification.

In Europe, the most encountered productive respiratory diseases include Ovine Respiratory Complex (ORC), Ovine Pulmonary Adenocarcinoma (OPA), verminous pneumonia caused by parasitic lungworms, and gangrenous (aspiration) pneumonia. These conditions typically present with pronounced clinical signs such as dyspnoea, a productive cough, and characteristic abnormal pulmonary sounds detectable on auscultation, including crackles, wheezes, and rhonchi. In contrast, non-productive respiratory diseases of concern include the pulmonary form of small ruminant lentivirus infection (SRLV) and the visceral form of caseous lymphadenitis (CLA). These conditions generally produce more subtle clinical signs, such as dyspnoea, mild to moderate dry cough, and occasionally wheezes audible on auscultation [7].

ORC represents a globally prevalent bacterial syndrome affecting sheep of all ages, historically referred to as “*pasteurellosis*” due to the predominant role of bacteria from the Pasteurellaceae family. It is also known as enzootic or atypical pneumonia [5]. ORC is a multifactorial disease resulting from a complex interaction between infectious agents and the host’s immune system, often compromised by environmental stressors and management practices. Notably, causative bacteria are often part of the normal respiratory microbiota, becoming pathogenic under certain conditions [9]. ORC stands as the leading cause of mortality and pulmonary lesions in feedlot lambs [10], as well as a major cause of respiratory disease in adult sheep [11]. Furthermore, it ranks as the second most common respiratory disease in dairy ewes [12]. The clinical spectrum of ORC is broad: peracute cases are often linked to *Bibersteinia trehalosi*, acute cases to *Mannheimia haemolytica*, and chronic infections frequently involve *Pasteurella multocida* and *Mycoplasma* sp. In view of mixed infections are common in these pneumonias [13]. The hallmark chronic lesion associated with ORC is pulmonary consolidation with a reddish-brown hue, predominantly affecting the cranioventral lung lobes, consistent with chronic bronchopneumonia [13,14].

OPA is a contagious neoplastic lung disease caused by the Jaagsiekte sheep retrovirus (JSRV), which induces malignant transformation of secretory epithelial cells in the alveoli and bronchioles, resulting in extensive pulmonary involvement [15]. To date, no widely effective preclinical diagnostic tests, vaccines, or control strategies have been successfully developed for the management of OPA [16].

Gangrenous pneumonia is a severe and often fatal condition affecting sheep of all ages, particularly lambs reared on artificial milk and adults prone to accidental aspiration of plant material [11,17]. The disease typically arises from the introduction of foreign material contaminated with *Trueperella pyogenes*. Other bacteria, such as *Pasteurella multocida* and *Mesomycoplasma ovipneumoniae*, which are normally part of the respiratory microbiota, act opportunistically to colonise the lesion and exacerbate pulmonary damage. These organisms are among the most frequently isolated from affected animals [5]. The characteristic pathological finding is extensive pulmonary necrosis with foul-smelling exudate that can extend into the major airways, significantly worsening the prognosis [18].

SRLV infection is a widespread viral disease affecting sheep and goats worldwide, with Europe reporting the highest individual prevalence [19]. SRLV includes both Caprine Arthritis Encephalitis Virus (CAEV) and Maedi-Visna Virus (MVV), which share phylogenetic and clinical similarities and can cross species barriers. In sheep, the pulmonary form typically presents as chronic interstitial pneumonia characterised by progressive respiratory compromise, weakness, and weight loss [20]. This disease imposes considerable economic burdens through reduced productivity and increased management costs [21].

CLA, caused by *Corynebacterium pseudotuberculosis*, is a chronic, contagious bacterial infection responsible for weight loss, reduced productivity, and culling-related economic losses worldwide [22]. Involvement of the respiratory system, particularly the mediastinal lymph nodes and lung tissue, accounts for approximately 85% of lesions identified in slaughtered sheep [23]. Because these lesions are mainly located within the thoracic cavity, they often give rise to respiratory signs that closely resemble those observed in ovine pulmonary lentivirus infections, thereby complicating the clinical diagnosis. Affected lymph nodes typically exhibit marked enlargement with thick, greenish pus surrounded by fibrous tissue, often displaying the characteristic ‘onion ring’ layering pattern on cross-section [24].

Verminous pneumonia, a parasitic lung disease caused primarily by *Dictyocaulus filaria*, *Protostrongylus rufescens*, and *Muellerius capillaris*, is endemic in many sheep populations. Many infected animals remain asymptomatic, making field diagnosis challenging [25]. The disease is characterised by focal granulomatous eosinophilic pneumonia predominantly affecting the diaphragmatic lobes, areas critical for effective ventilation [26]. Its high subclinical prevalence is reflected by the frequent pulmonary lesions observed at slaughter [27].

Given the wide range and overlapping clinical features of LRDs in sheep, in vivo diagnostic techniques capable of accurate differentiation are essential. Serological assays are mainly limited to SRLV infections, whereas coprological examinations remain the only diagnostic tool for verminous pneumonia [7]. Consequently, imaging modalities such as ultrasonography, radiography, and computed tomography have become indispensable for the diagnosis of LRDs [28].

Computed tomography (CT) provides unmatched detail in evaluating the lower respiratory tract of small ruminants, enabling the detection of lesions and anatomical abnormalities often missed by conventional diagnostic methods. For the upper respiratory tract, CT has demonstrated a remarkable 95% concordance with post-mortem findings, far surpassing the 68% concordance achieved with traditional imaging techniques [29]. Moreover, CT allows the creation of high-resolution digital models, enhancing research, diagnostic accuracy, and treatment planning in veterinary medicine [30]. Despite practical limitations related to cost, the need for anaesthesia, and radiation exposure, CT remains invaluable for animals of high genetic merit, experimental subjects, and exotic species.

This case-based study aims to provide a comprehensive analysis of CT imaging findings in sheep with LRDs, assessing both the diagnostic accuracy and clinical applicability of this advanced imaging modality in veterinary practice. Our objective is to improve the understanding and management of respiratory diseases, thereby enhancing animal welfare and productivity in the sheep industry. Furthermore, the detailed characterisation of radiological patterns associated with specific pathologies, combined with quantitative assessments using Hounsfield units, offers deeper insight into the underlying pathophysiology. This approach enables more precise differential diagnoses and supports evidence-based decision-making in clinical settings.

## 2. Materials and Methods

### 2.1. Animals and Inclusion Criteria

Between June 2017 and December 2024, 58 adult female sheep were enrolled through the Ruminant Clinical Service (SCRUM) at the University of Zaragoza. The animals originated from 8 farms in Aragón, the primary referral area for SCRUM. These farms operated under semi-intensive systems, with housing provided during critical physiological stages such as parturition and lactation.

Each animal underwent a standardised clinical diagnostic protocol that included a complete clinical examination, with particular emphasis on the respiratory system via auscultation. The initial step in evaluating the respiratory tract involved inducing coughing to determine whether it produced a moist or productive sound, thereby guiding the differential diagnosis toward conditions associated with secretions. During auscultation, additional findings such as rhonchi, crackles, and wheezes were recorded. However, the presence of a dry or non-productive cough did not necessarily exclude a productive process, as prior expectoration of mucus could have eliminated abnormal sounds. In cases of true non-productive pathology, wheezing was typically the only abnormal sound detected. If clinical signs suggested a productive respiratory process, the “wheelbarrow test” was performed to confirm or rule out OPA [7]. The odour of the cough was also evaluated to assist in the diagnosis of gangrenous pneumonia, as a characteristic putrid smell of the exhaled air can be detected due to the presence of necrotising exudates in the lungs [7]. When both conditions were excluded, a presumptive diagnosis of ORC was established. In contrast, dry and diffuse pulmonary lesions raised suspicion for SRLV infection. Because CLA may mimic SRLV or remain clinically silent, it was also included in the differential diagnosis.

Although these respiratory diseases represent the most common pulmonary pathologies in sheep, less frequent lesions, including tuberculosis or hydatid cysts, were also considered. Based on the initial clinical findings, additional in vivo diagnostic procedures, including CT scan, were performed to establish a presumptive diagnosis, with final confirmation obtained through post-mortem examination.

### 2.2. CT Imaging Protocol

CT scans were performed using a Brivo CT385 dual-slice scanner (GE Healthcare, Chicago, IL, USA) in helical mode. Each sheep underwent a single scan comprising two series (soft tissue and bone window algorithms), as well as a specific airway protocol. Imaging parameters were standardised at 80 mA and 120 kV. Multiplanar reconstructions (MPRs) were also performed as required, based on radiological criteria and case-specific needs.

Anaesthesia was induced intravenously using dexmedetomidine hydrochloride (0.005 mg/kg; Dexdomitor^®^ 0.5 mg/mL, Zoetis España, Madrid, Spain) and buprenorphine hydrochloride (0.01 mg/kg; Vetergesic^®^ 0.3 mg/mL, Ceva Salud Animal, Barcelona, Spain), followed by titrated propofol (1 mg/kg IV, adjusted to effect; Propofol Lipuro^®^ 10 mg/mL, B. Braun Medical S.A., Melsungen, Germany). Once the desired anaesthetic depth was achieved, animals were intubated and positioned in sternal recumbency. Anaesthesia was maintained using 2% isoflurane in oxygen (IsoFlo^®^, Zoetis España, Madrid, Spain). Given the underlying airway involvement, special care was taken to ensure adequate ventilation and airway patency throughout the imaging process.

The scan range extended from the thoracic inlet to the diaphragm. Animals were immobilised using non-radiopaque straps and plastic supports to minimise movement artefacts. Image review and interpretation were conducted using RadiAnt DICOM Viewer v4.6.9 (Medixant, Poznań, Poland), and radiological reports were generated describing lesion location, distribution, attenuation characteristics, and impact on surrounding structures.

To enhance the differentiation and visualisation of pulmonary pathologies using the RadiAnt DICOM Viewer software (version 2024.1; Medixant, Poznań, Poland), the window width (WW) was adjusted to the CT Lungs setting, which displays the image in a greyscale corresponding to a window level (WL) of −400 and a window width (WW) of 1500. To measure the Hounsfield units (HUs) of the lesions, each lesion was first located and then assessed using the “Ellipse” tool, which provides the mean HU value for the selected area. For greater accuracy, the smallest possible regions were selected, typically ranging between 0.02 and 0.04 cm^2^. In each lung analysed, 10 measurements were taken from different points, and the average HU was calculated accordingly. Hounsfield Units (HUs), also known as CT numbers, represent tissue density on a standardised greyscale in computed tomography. The HU scale ranges from +1000 for cortical bone, 0 for water, to −1000 for air, with all other tissues distributed along this scale in varying shades of grey [31]. Prior to examining pathological areas, data were collected from healthy pulmonary regions in the lungs of six healthy sheep using this CT scanner and methodology to establish baseline HU values. The results revealed that normal lungs exhibited mean HU readings between −550 and −850, consistent with those reported by other authors for a healthy, normally aerated lung in adult sheep [32,33].

### 2.3. Post-Mortem Examination

Following the completion of CT imaging, and for humanitarian reasons related to their poor general condition, all animals were euthanised in accordance with ethical guidelines. Full necropsy examinations were performed by the Histology and Pathological Anatomy Unit of the Department of Animal Pathology at the University of Zaragoza, with particular focus on the lower respiratory tract and adjacent thoracic structures. Macroscopic findings were documented, and representative tissue samples were collected for further laboratory analyses, including histopathology, molecular testing, and microbiological examination. Photographic records were obtained for all affected lungs. Gross post-mortem findings were then integrated with laboratory results to establish definitive diagnoses, which were subsequently compared with CT findings to evaluate diagnostic accuracy.

#### 2.3.1. Histopathology

Suspected tissue samples were fixed in 10% neutral buffered formalin (NBF) for a minimum of 48 h at room temperature to ensure adequate tissue preservation. Following fixation, samples were dehydrated through graded alcohols, cleared in xylene, and embedded in paraffin using standard histological procedures. Tissue sections of 4 µm thickness were obtained using a rotary microtome and subsequently stained with Carazzi’s haematoxylin and eosin (Bio-Optica Milano S.p.A., Milan, Italy) following conventional protocols. Histological evaluations were carried out by board-certified pathologists from the Histology and Pathological Anatomy Unit, Department of Animal Pathology, University of Zaragoza, who provided diagnostic confirmation. In conditions such as verminous pneumonia and pulmonary hydatidosis, histological examination was crucial, as it served as the sole definitive diagnostic tool in the absence of specific aetiological tests.

#### 2.3.2. Pathogen Detection

All 58 cases were screened for infectious agents commonly associated with lower respiratory tract disease. Lung tissue samples were analysed using real-time PCR (qPCR) to detect the DNA of *Mesomycoplasma ovipneumoniae*, *Bibersteinia trehalosi*, *Mannheimia haemolytica*, *Pasteurella multocida*, the OPA virus, and SRLV. In cases with suspected caseous lymphadenitis, identification of *Corynebacterium pseudotuberculosis* was also performed and confirmed either by microbiological culture or by qPCR following lesion swabbing. All samples were analysed according to routine diagnostic protocols at the same private laboratory, EXOPOL (Zaragoza, Spain).

### 2.4. Statistical Analysis

Descriptive statistics were used to characterise the study population and diagnostic outcomes. Sensitivity (TP: true positives)/[TP + FN: false negatives] and specificity (TN: true negatives)/[TN + FP: false positives] were calculated to evaluate diagnostic performance. Diagnostic accuracy and agreements between diagnostic techniques were evaluated using Cohen’s kappa statistic (κ) as follows: no agreement (κ < 0), slight agreement (0 < κ < 0.2), fair agreement (0.2 < κ < 0.4), moderate agreement (0.4 < κ < 0.6), substantial agreement (0.6 < κ < 0.8), and almost perfect agreement (κ > 0.8). All statistical analyses were conducted using IBM SPSS Statistics version 28.0 (IBM Corp., Armonk, NY, USA).

## 3. Results

### 3.1. Case Distribution

The animals included in this study originated from farms located in the Aragón region of northeastern Spain. A total of 58 female sheep were evaluated, consisting of 47 Rasa Aragonesa (81.0%), 4 Merino (6.9%), and 7 mixed-breed individuals (12.1%).

### 3.2. Clinical Findings

A total of 58 female sheep presented to the Faculty of Veterinary Medicine were examined, and a preliminary clinical classification was established for each case based solely on clinical signs. The categories included apparently healthy animals (*n* = 26), cases compatible with ORC (*n* = 21), interstitial pneumonia associated with SRLV infection (*n* = 6), gangrenous infections (*n* = 4), OPA (*n* = 3), and verminous pneumonia (*n* = 1). No animals exhibited clinical signs suggestive of CLA or hydatidosis at this stage. It should be noted that animals considered clinically healthy could still harbour pulmonary lesions that were only detected at necropsy.

### 3.3. Tomographic Findings

Evaluation of the CT scans revealed a total of 82 lower respiratory tract lesions in the 58 sheep studied, as several animals presented with multiple conditions. Diagnoses were consistent with interstitial pneumonia (*n* = 29), gangrenous infections (*n* = 17), ORC (*n* = 16), CLA (*n* = 9), verminous pneumonia (*n* = 6), OPA (*n* = 3), and pulmonary hydatidosis (*n* = 2). No cases were classified as healthy based on CT findings (Table 1).

#### 3.3.1. Interstitial Pneumonia (SRLV)

**Figure 1:**
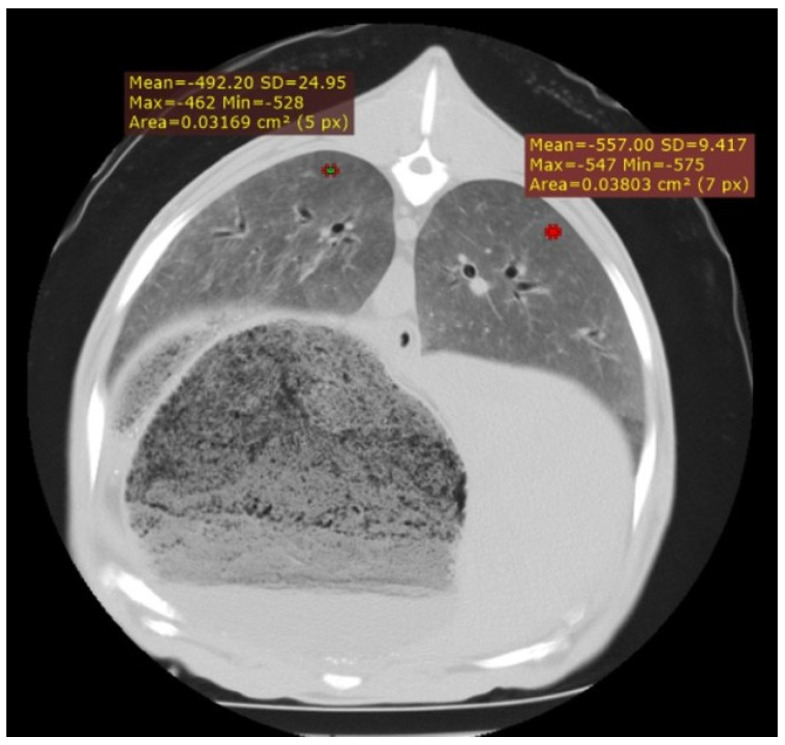
Cross-section of the thorax of an adult sheep affected by interstitial pneumonia, showing a diffuse increase in lung parenchyma density corresponding to increased cellularity in the pulmonary interstitium. In addition, two HU measurement points are displayed.

In the 29 cases of suspected interstitial pneumonia associated with SRLV, a variable increase in pulmonary density was observed, diffusely affecting the entire lung parenchyma. This increase in density corresponded to elevated cellularity within the pulmonary interstitium (Figure 1) and was more pronounced in animals at advanced stages of the disease. To quantify this involvement, HU measurements were taken from the dorsal lung regions in a subset of randomly selected animals. Values ranged from −400 to −800 HU, with this variability reflecting the degree of cellular infiltration. In the most severely affected cases, characterised by extensive pulmonary involvement and high cellularity, values ranged from −400 to −550 HU; in moderately affected cases, from −550 to −700 HU; and in milder cases, with minimal pulmonary compromise, from −700 to −800 HU.

#### 3.3.2. Gangrenous Pneumonia (GN)

**Figure 2:**
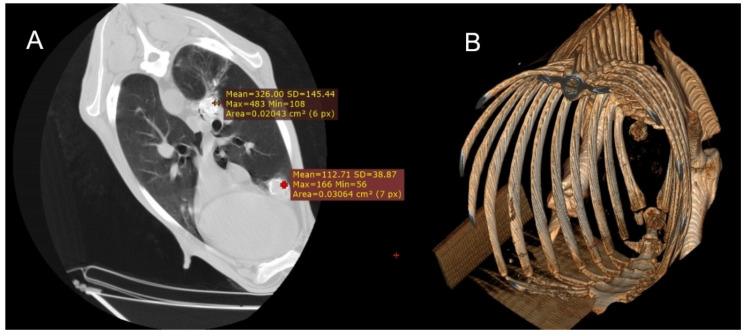
(**A**) Transverse section of the thorax of a sheep showing two foci of calcified gangrenous pneumonia, with Hounsfield Units (HUs) measured at both sites. (**B**) Three-dimensional reconstruction of the same animal, where after isolating only the skeletal regions, the calcified areas of the lung become visible.

In the CT examination of the six animals suspected of gangrenous or aspiration pneumonia, pulmonary lesions were primarily located in the cranioventral lobes, although extension into adjacent lobes was common as the disease progressed. These affected regions were characterised by hypodense areas consistent with necrotic tissue, displaying poorly defined margins and progressive loss of normal pulmonary architecture. In advanced cases, cavitations lacking air content were observed, indicating parenchymal destruction and complete loss of function in these regions. Occasionally, these necrotic cavities contained purulent material, sometimes with evidence of calcification (Figure 2), and in some cases, aspirated material carrying the aetiological agent was detected. HU measurements taken from the cavity walls ranged from 20 to 50 HU; however, when the intralesional content was purulent or putrid, values increased substantially, reaching 150–600 HU in the centre of the necrotic cavities.

#### 3.3.3. Chronic Form of Ovine Respiratory Complex (ORC)

**Figure 3:**
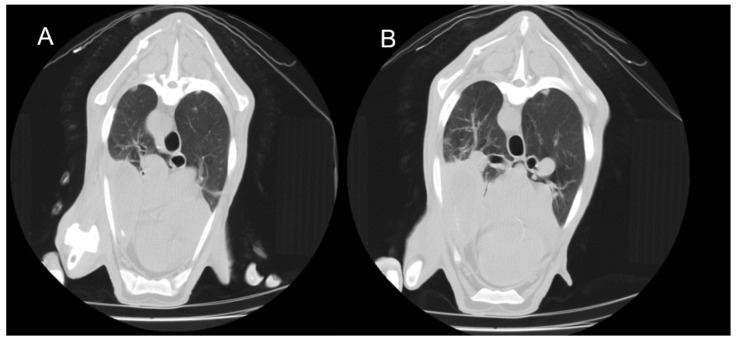
Transverse thoracic CT slices of two adult sheep showing consolidation of the right cranioventral lung lobe. (**A**) Caudal thoracic slice. (**B**) More caudal thoracic slice. The consolidated area appears denser than the surrounding aerated lung regions.

**Figure 4:**
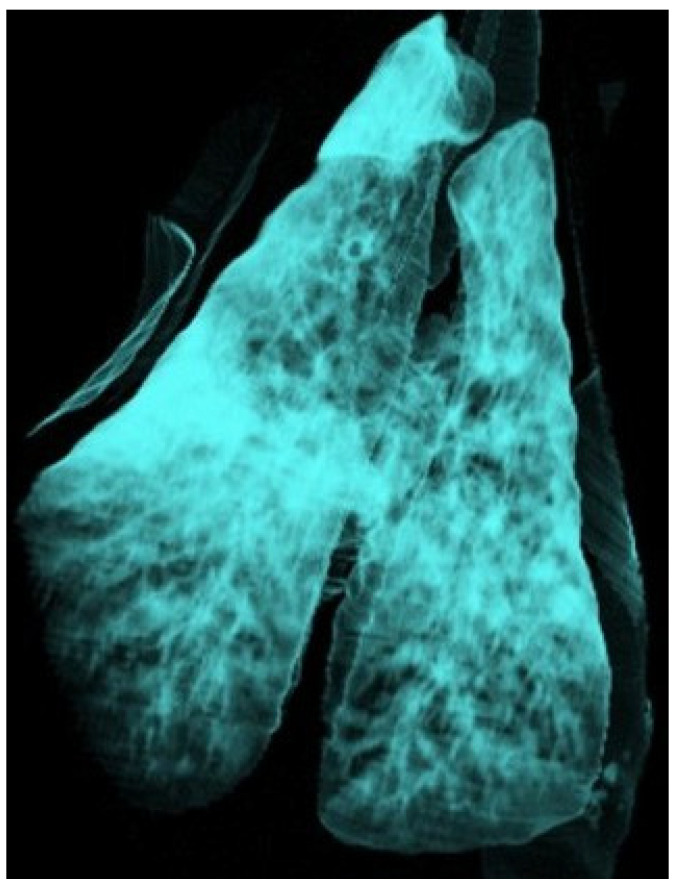
Three-dimensional reconstruction of the lung of a sheep using the airway algorithm, with aerated regions represented in blue. The lung shows a diffuse reduction in colour intensity consistent with interstitial pneumonia, as well as a consolidated cranioventral area compatible with ovine respiratory complex (ORC).

In the 16 animals with CT findings compatible with the chronic form of ORC, lobar consolidations with complete collapse of portions of the lung parenchyma were observed. These affected regions appeared as hyperdense, airless areas with associated volume loss, resulting in distortion of the pulmonary silhouette (Figure 3). Fully collapsed pulmonary regions were consistently visualised as denser (whiter) than adjacent functional lung tissue. Bronchiectasis was occasionally present within these areas, indicating chronic structural damage to the respiratory parenchyma (Figure 4). HU measurements were obtained from multiple representative points within the lesions, particularly along the lateral regions near the ribs. Recorded values typically ranged from 40 to 60 HU, consistent with increased tissue density associated with alveolar collapse and parenchymal remodelling.

#### 3.3.4. Caseous Lymphadenitis (CLA)

**Figure 5:**
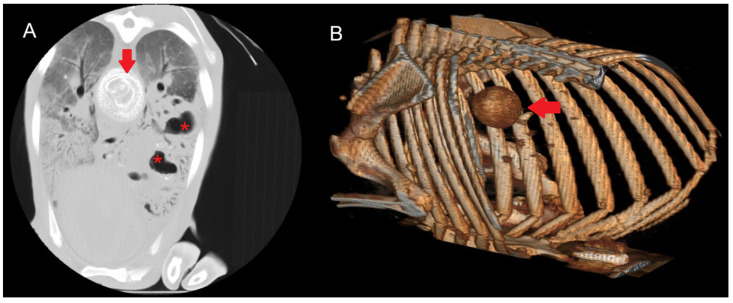
(**A**) Transverse section of a sheep lung showing a calcified mediastinal lymph node (arrow) due to CLA. The characteristic concentric layers of this type of caseous necrosis are visible, together with areas of increased density and cavitations (red asterisk) consistent with gangrenous pneumonia. A diffuse increase in lung parenchyma density corresponding to interstitial pneumonia is observed. (**B**) Three-dimensional reconstruction showing the calcified mediastinal lymph node.

In the six cases of suspected CLA, rounded hyperdense structures were observed. In some animals, involvement of the surrounding tissue was also noted, along with areas of mineralisation (Figure 5). These lesions were located in the mediastinal lymph nodes, the pulmonary parenchyma, or both.

HU measurements were obtained from both the outer, non-calcified portion of the lymph node and the central region when calcification was present, allowing for direct comparison. Values in the non-calcified external region ranged from 10 to 60 HU, whereas centrally calcified areas recorded values between 150 and 600 HU. In lesions with developing calcification, intermediate values between 100 and 200 HU were observed.

#### 3.3.5. Verminous Pneumonia (VP)

**Figure 6:**
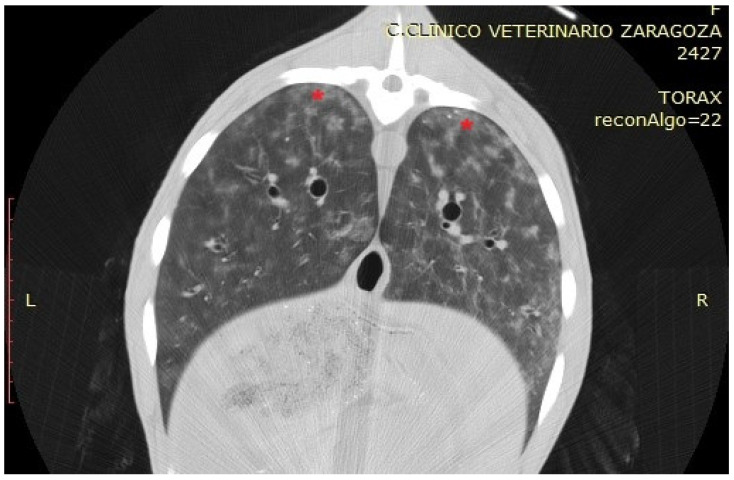
Transverse section of a sheep showing, with red asterisks, an area corresponding to nodules of verminous pneumonia in the dorsal part of the diaphragmatic lobes.

In the CT examination, pulmonary lesions associated with parasitic infestation appeared as areas of tissue thickening with increased density, presenting as hyperdense regions within the affected zones. These lesions displayed a characteristic anatomical distribution: the caudal and diaphragmatic areas were typically associated with *Dictyocaulus* infestations, whereas the dorsal regions of the pulmonary parenchyma were more commonly affected in *Protostrongylus* infections. Granulomatous pneumonia was characterised by multiple well-defined nodules predominantly located in the dorsocaudal lung regions, exhibiting a focal distribution pattern without diffuse parenchymal involvement (Figure 6). To evaluate tissue density, HU measurements were obtained from the centre of the hyperdense areas in two specimens. Values ranged from −100 to −300 HU, consistent with partially aerated tissue or inflammatory infiltrates rather than complete consolidation, suggesting an intermediate stage of the chronic inflammatory process induced by parasitic infestation.

#### 3.3.6. Ovine Pulmonary Adenocarcinoma (OPA)

**Figure 7:**
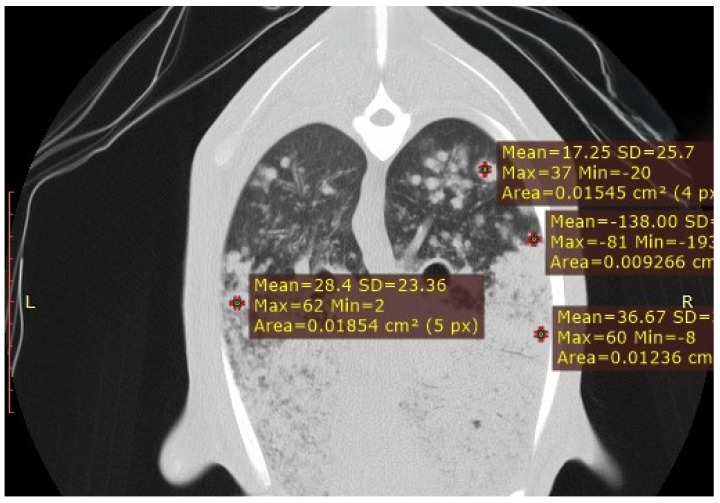
CT transverse section of a sheep lung showing multiple satellite nodules of varying sizes surrounding the primary tumour mass. HU measurements were obtained from both the outer region of the primary tumour and the satellite nodules, highlighting differences in tissue composition between the primary and metastatic lesions.

**Figure 8:**
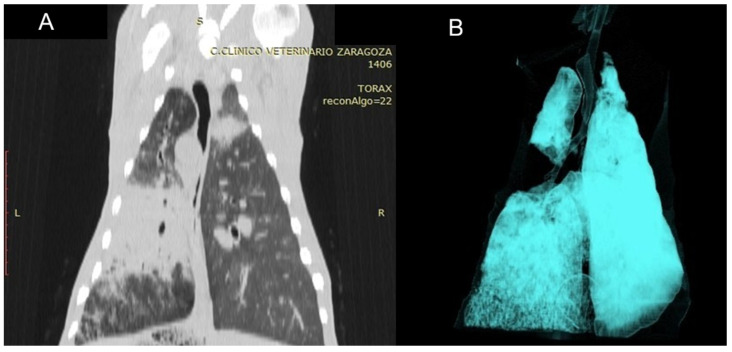
(**A**) Dorsal CT section of the thorax of a sheep affected by OPA, showing the primary tumour mass in the left lung together with satellite masses, visible as areas of increased tissue density. (**B**) Three-dimensional reconstruction using the airway algorithm from the same animal, demonstrating complete absence of ventilation in the central lung region.

In the three cases of suspected OPA, solid masses with irregular margins and homogeneous density were identified, resulting in focal consolidations (Figure 7). Multiple satellite nodules of varying sizes were occasionally detected surrounding these masses, corresponding to local metastases originating from the primary tumour (Figure 8). To radiologically characterise these lesions, HU measurements were obtained from both the outer region of the primary tumour and the satellite nodules. The peripheral tumour area showed values ranging from −5 to −30 HU, whereas the metastatic regions recorded values between −90 and −200 HU, indicating differences in tissue composition between the primary and secondary lesions.

#### 3.3.7. Pulmonary Hydatidosis (PH)

**Figure 9:**
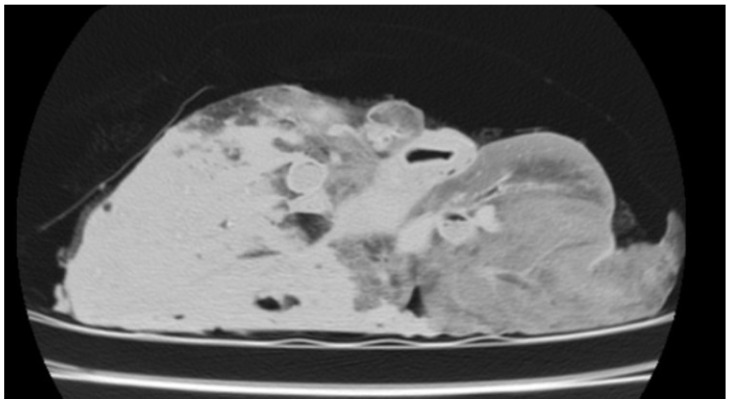
CT image of the isolated lung from a slaughtered sheep, showing cystic regions consistent with pulmonary hydatidosis.

Two cases were included in this study based on CT findings consistent with pulmonary hydatidosis, characterised by well-defined cystic lesions with regular borders and homogeneous content. These lesions were typically located in the lower lobes and were multiple in both cases, occurring either unilaterally or bilaterally. The cysts displayed a rounded or oval morphology with thin, smooth walls. Lesions at different stages of the life cycle were commonly observed; some showed calcification, leading to increased density and a more hyperdense appearance on CT. HU measurements obtained from the outermost region of the cystic lesions ranged from −50 to −75 HU, values consistent with the liquid-cystic nature of the content and the characteristic tissue composition of these lesions (Figure 9).

The Hounsfield Unit (HU) values associated with the different pulmonary lesions are summarised in Table 1.

### 3.4. Post-Mortem Findings

Among the 58 animals included in this study, the following conditions were diagnosed: interstitial pneumonia associated with SRLV (*n* = 32), ORC (*n* = 22), GP (*n* = 17), CLA (*n* = 10), VP (*n* = 9), OPA (*n* = 2), and PH (*n* = 2). No animals were found to be free of pulmonary lesions (*n* = 0).

#### 3.4.1. Histopathological Findings

A total of 11 lung samples with lesions were evaluated, confirming VP (*n* = 9) and PH (*n* = 2).

#### 3.4.2. Pathogen Detection

Lung tissue samples were subjected to qPCR and microbiological culture to detect the principal pulmonary pathogens affecting sheep. In cases of interstitial pneumonia (*n* = 32), SRLV was consistently identified. In OPA cases (*n* = 2), the causative virus, JSRV, was detected, whereas *Corynebacterium pseudotuberculosis* was found in all animals with lesions consistent with CLA (*n* = 10).

As expected for these conditions, cases of ORC (*n* = 22) and GP (*n* = 17) frequently involved multiple bacterial pathogens. Among these, five animals (20.8%) exhibited coinfections with between one and three distinct agents per case. In the 17 animals affected exclusively by ORC, the most frequently detected pathogens were *Mannheimia haemolytica* (MH) in 15 cases (88.2%), *Mesomycoplasma ovipneumoniae* (MO) in 12 (70.6%), and *Pasteurella multocida* (PM) in 11 (64.7%), while *Trueperella pyogenes* (TP) was not detected.

In contrast, among the 12 animals affected exclusively by gangrenous pneumonia, TP was the predominant pathogen, detected in 8 animals (66.7%) and frequently co-detected with PM (6 animals, 50.0%). Across all 24 cases of ORC and gangrenous pneumonia (including both exclusive and coinfected presentations), the most common bacterial combination was PM + MO + MH (9 animals, 37.5%), followed by coinfections involving all four agents (TP + PM + MO + MH, 5 animals, 20.8%). Other profiles included TP + PM (4 animals, 16.7%), isolated PM or TP (2 animals each, 8.3%), and less frequent patterns such as PM + MO or PM + MH (1 animal each, 4.2%).

#### 3.4.3. Statistical Analysis

The degree of agreement between clinical evaluation, computed tomography (CT), and post-mortem findings was assessed across several pulmonary diseases using Cohen’s Kappa coefficient (κ) as a measure of inter-rater reliability beyond chance (Table 2).

Clinical evaluation demonstrated low concordance with post-mortem diagnoses, with κ values ranging from −0.03 to 0.45. The lowest levels of agreement were observed for SRLV (κ = −0.02), VP (κ = −0.03), PH (κ = 0.00), and CLA (κ = 0.00). The highest agreement based on clinical assessment was for ORC (κ = 0.45).

In contrast, CT showed substantially higher concordance with post-mortem findings across most conditions. Almost perfect agreement was achieved for PH (κ = 1.00), CLA (κ = 0.94), GP (κ = 0.92), and SRLV (κ = 0.90). Substantial agreement was observed for VP (κ = 0.77) and ORC (κ = 0.61). For OPA, both clinical evaluation and CT demonstrated only moderate agreement with post-mortem diagnoses (κ = 0.37).

It is important to note that some conditions, particularly PH and OPA, had very small sample sizes, and therefore the observed differences in concordance should be interpreted with caution, as they may not be statistically significant. Overall, these findings indicate that CT provides a more reliable assessment of pulmonary lesions than clinical evaluation, especially for conditions with larger sample sizes.

## 4. Discussion

This study demonstrates the usefulness of CT scanning in the accurate diagnosis of LRDs in adult sheep. It highlights the broad spectrum of respiratory pathologies frequently included in the differential diagnosis of LRDs, many of which are clinically indistinguishable due to overlapping signs and subclinical presentations [7]. The prevalence of conditions such as ORC and interstitial pneumonia associated with SRLV observed here aligns with previous reports [5,34], reaffirming their economic significance and health impact on the global sheep industry. These diseases not only contribute to mortality but also reduce productivity and increase carcass condemnation rates at slaughter [21,35]. Furthermore, hydatidosis, although less common, emerges as a relevant zoonosis with substantial economic implications and a notable public health concern, particularly in endemic Mediterranean regions such as Spain [36]. While the liver remains the most frequent site of hydatid cyst development, severe infestations may also affect the lungs, as confirmed in this study.

The findings of this study strongly confirm the superior diagnostic accuracy of CT compared with conventional clinical evaluation. This is supported by the high concordance observed between CT-based diagnoses and post-mortem findings, a relationship previously documented in upper respiratory diseases [29]. Unlike radiography, CT can detect subtle or early-stage pulmonary lesions with significantly higher sensitivity [37,38], providing detailed visualisation of pulmonary changes that would otherwise remain undetected. Pulmonary ultrasonography also represents a highly useful and practical diagnostic tool, particularly for field applications; however, CT offers a much greater level of anatomical detail, allowing a more precise characterisation of lesions and improving the accuracy of the final diagnosis. Experimental evidence further supports its utility in identifying early interstitial lesions caused by viral agents such as SRLV or Jaagsiekte sheep retrovirus before the onset of clinical signs [39,40,41].

Moreover, CT has proven valuable in quantifying tumour progression and evaluating lung pathology in vivo using objective scoring systems [16,41], particularly in pulmonary cancer models [42]. Despite these diagnostic advantages, its clinical application in sheep remains limited due to high costs, the requirement for general anaesthesia, and limited availability in rural or resource-constrained settings. Nevertheless, CT becomes indispensable in high-value cases such as elite genetic stock or animals with complex, inconclusive clinical profiles. In research contexts, CT scanning also plays a crucial role in experimental design and longitudinal disease monitoring [29,30,43].

By contrast, thoracic ultrasonography remains the preferred tool for routine detection of pulmonary pathology, owing to its strong correlation with post-mortem results [14]. However, its sensitivity is reduced for detecting subtle or deep parenchymal lesions. A particularly novel contribution of this study is the standardisation of HU measurements across various LRDs in sheep. Preclinical research in animal models has demonstrated that HU quantification serves as a robust marker of structural pulmonary changes [44,45]. The variability in HU values observed here reflects important differences in tissue density, consistently correlating with histological and functional alterations in both small-animal micro-CT and large-animal CT studies [46,47]. This study therefore provides one of the first comprehensive descriptions linking CT-based pathology with HU quantification in sheep, reinforcing the species’ potential as an experimental model for respiratory disease research [48].

Importantly, up to three concurrent respiratory pathologies were identified within individual animals, underscoring both the inherent complexity of respiratory disease in sheep and the limitations of clinical examination alone. CT scanning proved instrumental in detecting coexisting lesions, mixed attenuation patterns, and heterogeneous anatomical distributions that would otherwise remain undetected. Similar findings of multiple concurrent infections, such as OPA, SRLV infection, and the ORC, have been reported previously [49,50,51], further highlighting the diagnostic challenges and management implications of such presentations.

At present, CT is best regarded as a valuable research tool for investigating and monitoring respiratory diseases in sheep. Its ability to provide detailed, three-dimensional visualisation of pulmonary structures makes it particularly useful for studying the evolution of conditions such as OPA and SRLV-associated pneumonia, or for validating other diagnostic techniques. Future technological advances—such as portable or lower-cost systems—may eventually broaden its applicability, but its current relevance lies primarily in improving understanding of disease processes and supporting the development of more effective diagnostic and preventive strategies within the framework of precision livestock farming.

Moreover, CT’s proven diagnostic utility in zoonotic diseases such as pulmonary hydatidosis [52], reinforces its broader relevance beyond animal health, addressing both economic and public health concerns. Taken together, these findings emphasise the limitations of traditional diagnostic methods such as auscultation in differentiating LRDs in sheep [53], particularly when multiple infections coexist. This highlights the growing need for imaging-based diagnostic strategies [14,29,53].

CT consistently demonstrates superior concordance with necropsy findings [30], reinforcing its value as a key ante mortem diagnostic tool in complex or ambiguous cases [29,54]. However, certain conditions, particularly PH and OPA, were represented by small sample sizes; therefore, differences in concordance should be interpreted with caution, as they may not be statistically significant. Overall, the results indicate that CT provides a more accurate and reliable assessment of pulmonary lesions than conventional clinical evaluation, especially for diseases with sufficient representation in the dataset. Importantly, this study also confirms the high prevalence of concomitant respiratory disorders in sheep, which often obscure the primary pathology and challenge clinical interpretation. By enabling objective, sensitive, and detailed visualisation of pulmonary structures, CT emerges as a decisive diagnostic resource that enhances early detection, refines differential diagnosis, and ultimately improves the understanding and management of respiratory disease in sheep.

## Figures and Tables

**Table 1:** HU ranges observed in different pulmonary lesions in sheep. HU measurements were obtained from representative areas of the lesions using computed tomography. Values reflect differences in tissue density, ranging from hypodense (air- or fluid-filled) to hyperdense (cellular infiltration, consolidation, or calcification) regions, corresponding to the severity and nature of each pathological condition.

Lesion/Disease	Location/Distribution	HU Range	Notes/Interpretation
SRLV	Diffuse, entire lung parenchyma	800 to −400 HU	Severity-dependent: mild (−700 to −800 HU), moderate (−550 to −700 HU), severe (−400 to −550 HU); reflects cellular infiltration
GN	Cranioventral lobes, may extend	20 to 50 HU (walls), 150 to 600 HU (purulent/necrotic content)	Hypodense necrotic tissue; high HU in purulent/putrid areas
Chronic ORC	Lobar consolidations, some collapsed regions	40 to 60 HU	Hyperdense, airless areas; alveolar collapse and parenchymal remodelling
CLA	Mediastinal lymph nodes, sometimes pulmonary parenchyma	10 to 60 HU (non-calcified), 100–200 HU (developing calcification), 150–600 HU (central calcification)	Rounded hyperdense structures; HU increases with calcification
VP	Caudal/diaphragmatic regions (*Dictyocaulus*), dorsal (*Protostrongylus*)	−100 to −300 HU	Hyperdense nodules; partially aerated tissue with inflammatory infiltrate
OPA	Primary masses with satellite nodules	−5 to −30 HU (primary), −90 to −200 HU (metastases)	Solid masses; differences in tissue composition between primary and metastatic lesions
PH	Lower lobes, unilateral/bilateral	−50 to −75 HU	Well-defined cysts with liquid content; calcified cysts show higher HU

**Table 2:** Cohen’s Kappa coefficient (κ) was used to assess the agreement between clinical evaluation, computed tomography (CT), and post-mortem findings across different pulmonary diseases. Abbreviations used: SRLV = small ruminant lentivirus; ORC = ovine respiratory complex; GP = gangrenous pneumonia; CLA = caseous lymphadenitis; VP = verminous pneumonia; OPA = ovine pulmonary adenocarcinoma; PH = pulmonary hydatidosis; CT = computed tomography.

Disease	Clinical Evaluation vs. Post-Mortem Finding		CT Evaluation vs. Post-Mortem Findings	
	Cohen’s Kappa Statistic (κ)	Agreement Interpretation	Cohen’s Kappa Statistic (κ)	Agreement Interpretation
SRLV	−0.02	No agreement	0.90	Almost perfect agreement
ORC	0.45	Moderate agreement	0.61	Substantial agreement
GP	0.30	Fair agreement	0.92	Almost perfect agreement
CLA	0	No agreement	0.94	Almost perfect agreement
VP	−0.03	No agreement	0.77	Substantial agreement
OPA	0.37	Fair agreement	0.37	Fair agreement
PH	0	No agreement	1	Almost perfect agreement

## Data Availability

The original contributions presented in this study are included in the article. Further inquiries can be directed to the corresponding author(s).
